# Successful Management of an Emerging Distinct Gingival Lesion With a New Histopathological Identity

**DOI:** 10.7759/cureus.71174

**Published:** 2024-10-09

**Authors:** Faris Almutairi

**Affiliations:** 1 Oral and Maxillofacial Surgery, College of Dentistry, Qassim University, Buraydah, SAU

**Keywords:** gingival fibroma, gingival growth, gingival hyperplasia, gingival hypertrophy, gingival neoplasms

## Abstract

Gingival fibroma is a pathological condition that can manifest in pediatric and adult patients, often presenting diagnostic challenges due to shared histopathological characteristics among various lesions. It has been reported as a novel category with distinct histopathological features with new diagnostic criteria. A 40-year-old Saudi female patient with no significant medical history reported a 4×3 cm pedunculated, non-tender, firm, red gingival growth adjacent to the remaining root of tooth number 27 in the maxillary left side. Radiographic assessment yielded remarkable findings. An excisional biopsy was performed, and histopathology analysis confirmed the lesion to be a gingival fibroma. This case marks the first reported instance of gingival fibroma in Saudi Arabia. The recognition of such distinctive histopathological features contributes to improved diagnostic accuracy and highlights the need for vigilance in identifying emerging pathological entities within the gingival overgrowths. The recognition of gingival fibroma as a unique pathological entity is clinically significant, resolving diagnostic challenges in gingival growth cases. Specific diagnostic criteria enable accurate differentiation from similar lesions. The advancement improves patient care and refines the understanding of gingival overgrowth pathogenesis.

## Introduction

Due to continuous exposure to diverse stimuli, the oral mucosa displays various conditions, from reactive alterations to tumorous formations [1]. Clinicians are consistently fascinated by the diverse presentation of gingival tissue, gingival growth being the most common observation. Gingival growth is often attributed to reactive hyperplasia, often stemming from inflammation caused by plaque-related gingival tissue [2]. Oral fibromas emerge from trauma to the underlying connective tissue, leading to tissue enlargement [3]. Surgical excision is often prescribed to mitigate ongoing trauma, with biopsies being recommended to confirm the diagnosis of these growths and exclude the possibility of malignancy [4].

In gingival pathology, the diagnostic term gingival fibroma (GF) has been used to characterize a distinct reactive gingival growth that exhibits distinctive histopathological attributes [5]. The term encapsulates the unique features observed in the previously unclassified condition. Gingival growths are predominantly reactive and seldom display notable true neoplastic propensity. The prevailing cause is local irritation from calculus, trauma, dental plaque, and medication-associated overgrowth [6]. When considering a differential diagnosis, it is essential to differentiate GF from other entities like peripheral giant cell granuloma (PGCG), periapical granuloma (PG), peripheral ossifying fibroma (POF), and peripheral odontogenic fibroma (POdF) [6].

Pyogenic granuloma is a common, non-cancerous vascular growth [7] that develops in tissues like the skin and mucous membranes, characterized by a single, red, fragile pedunculated papule most commonly found in the buccal region [7,8]. PG is the most prevalent benign overgrowth of the skin and oral mucosa, with a higher occurrence in females than males [9]. Its growth is normally limited to 2.5 cm, and recurrence rates are estimated at 14.8% [9,10]. POdF is an infrequent benign tumor originating from odontogenic cells, presenting as an exophytic mass with a smooth surface or oral mucosa [11]. It is commonly observed in the anterior mandible and can grow to sizes as substantial as 3-4 cm [1]. The peak incidence of this lesion is generally seen between the second and fourth decades of life. Surgical excision stands as the preferred treatment method [1]. POF is a mesenchymal benign lesion that exclusively affects the gingiva, presenting as a well-defined, asymptomatic, steadily enlarging, solid nodular tumor [12]. POF is more prevalent among female patients than males and predominantly affects the anterior maxilla [13]. PGCG primarily occurs in edentulous patients due to denture irritation, showing a higher incidence in females and a preference for the mandible. Clinically, it presents as a purplish-red vascular nodule, often with surface ulceration, commonly found on the gingiva or alveolar crest, especially near incisors and molars. Histopathologically, it displays numerous osteoclast-like multinucleated giant cells in a well-vascularized context, a hallmark feature distinguishing it from another reactive gingival tumor including the following features: prominent fibromyxoid background, storiform pattern cellular arrangement, almost no inflammation, endothelial proliferation, calcification, and epithelial odontogenic islands [6]. 

By providing detailed insights into the cases, the report aims to enhance the understanding of GF's varied manifestations, potential differential diagnoses, and effective treatment strategies. Additionally, the report seeks to contribute to the existing knowledge regarding GFs, enabling better clinical recognition, accurate diagnosis, and informed decision-making for optimal patient care.

## Case presentation

A 40-year-old Saudi female patient, devoid of comorbidities, medical ailments, or allergies, sought care in the Oral and Maxillofacial Department for a noticeable gingival swelling that started gradually six months back and localized on the left posterior maxillary side. Her medical history was unremarkable, with no comorbidities or significant medical history.

Clinical examination

**Figure 1:**
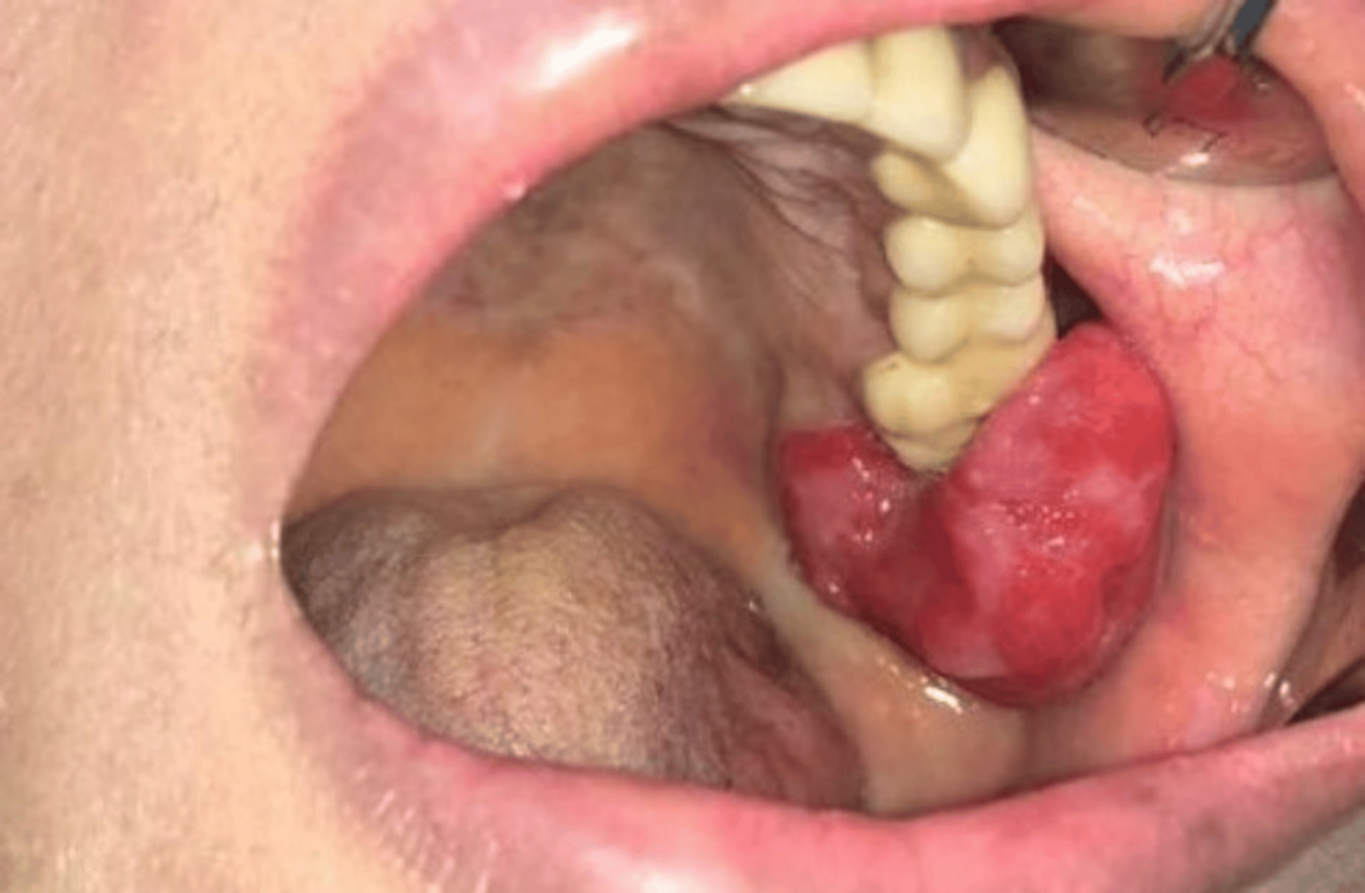
Clinical picture of the gingival overgrowth originating from the left side of the maxilla (tooth area 27)

The clinical examination revealed a large pedunculated growth measuring around 4×3 cm adjacent to the remaining root of tooth number 27 (FDI Numbering System) (Figure 1). The growth exhibited non-tender, firm, and red characteristics. The growth's evolution from a small nodule to its present size, extending both downward and buccally, was a notable clinical feature. The patient did not report any pain in the mass, nor did it bleed upon contact with an examination probe. The extra-oral examination, however, revealed slight asymmetry on the left lower cheek area due to swelling.

Radiographic examination

**Figure 2:**
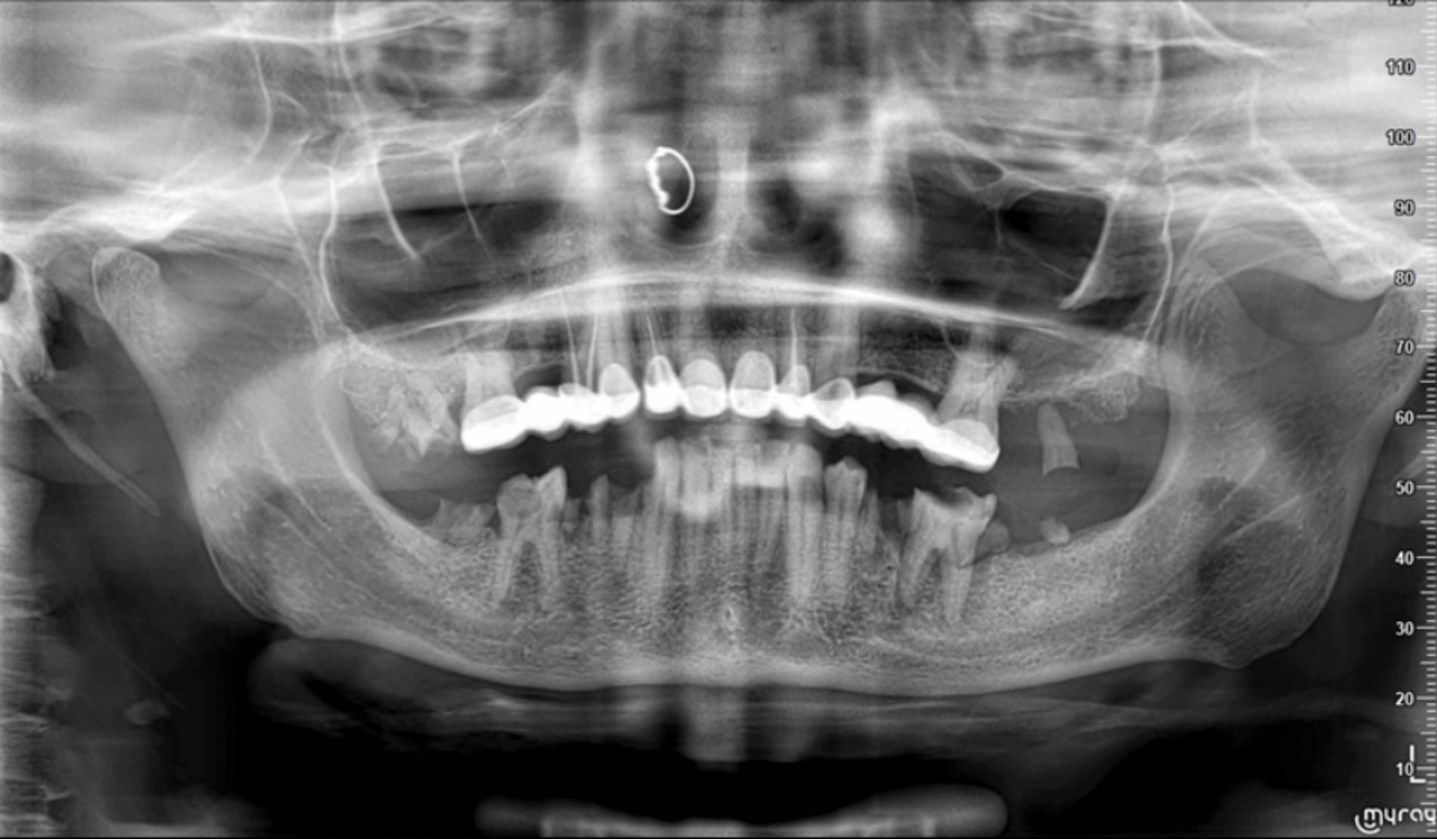
Panoramic X-ray showing the remaining root of tooth number 27

Upon radiographic examination (Figure 2), it was observed that there was no evidence of bone resorption or bone avulsion at the location of the gingival lesion. Additionally, the radiograph revealed the presence of the remaining tooth number 27, which appeared to be positioned almost entirely out of the bone. Apart from the findings related to gingival lesion and tooth number 27, the radiograph indicated the presence of other remaining roots and evidence of decayed teeth.

Histopathological examination

**Figure 3:**
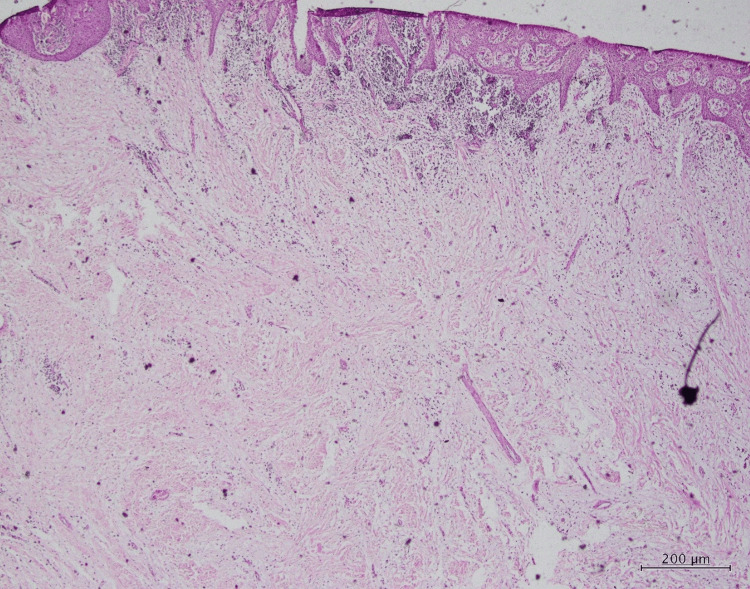
Low-power magnification of the histopathological slide: H&E section H&E: hematoxylin and eosin

**Figure 4:**
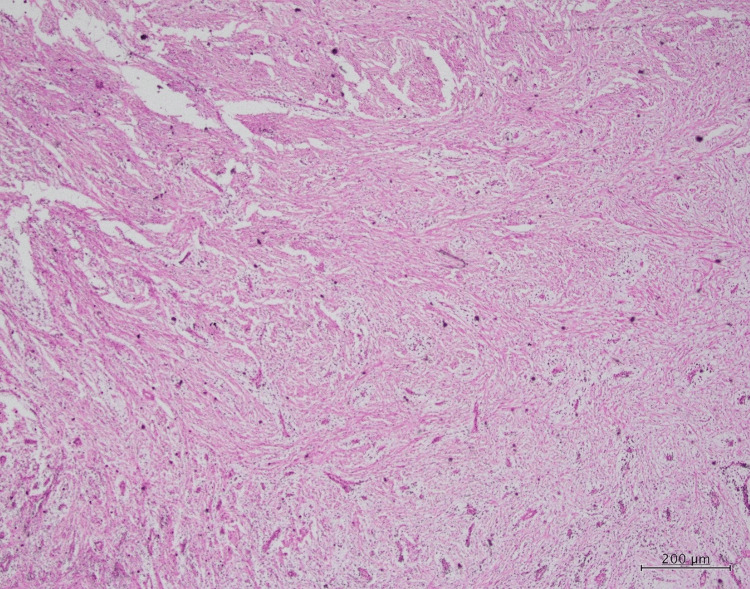
Medium-power (10×) magnification of the H&E section showing myxoid and fibrous tissues containing loose to dense collagen fibers, fibroblasts, and blood vessels H&E: hematoxylin and eosin

**Figure 5:**
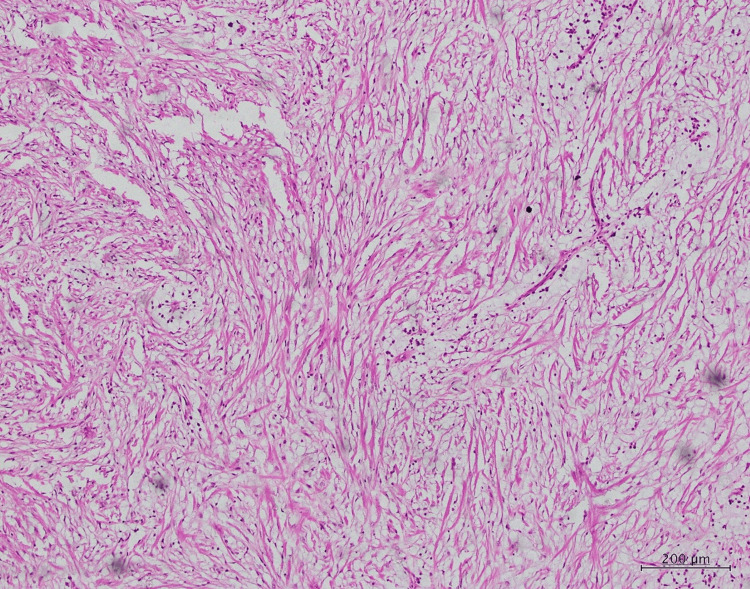
High-power (20×) magnification of the H&E section showing loosely arranged fibrous tissue H&E: hematoxylin and eosin

A meticulous analysis of the histopathological features was conducted, encompassing multiple levels of magnification (low power, medium power, and high power). Upon examination at a medium power of 10×, the hematoxylin and eosin (H&E)-stained pathological sections unveiled the presence of myxoid and fibrous tissues. These tissues exhibited an array of constituents, notably collagen fibers ranging from loose to dense. Fibroblasts and diversity in blood vessel sizes were prominently observed within this matrix, signifying the role of fibrotic processes and the vascular nature of the lesion (Figure 3, Figure 4, and Figure 5).

Treatment and follow-up

**Figure 6:**
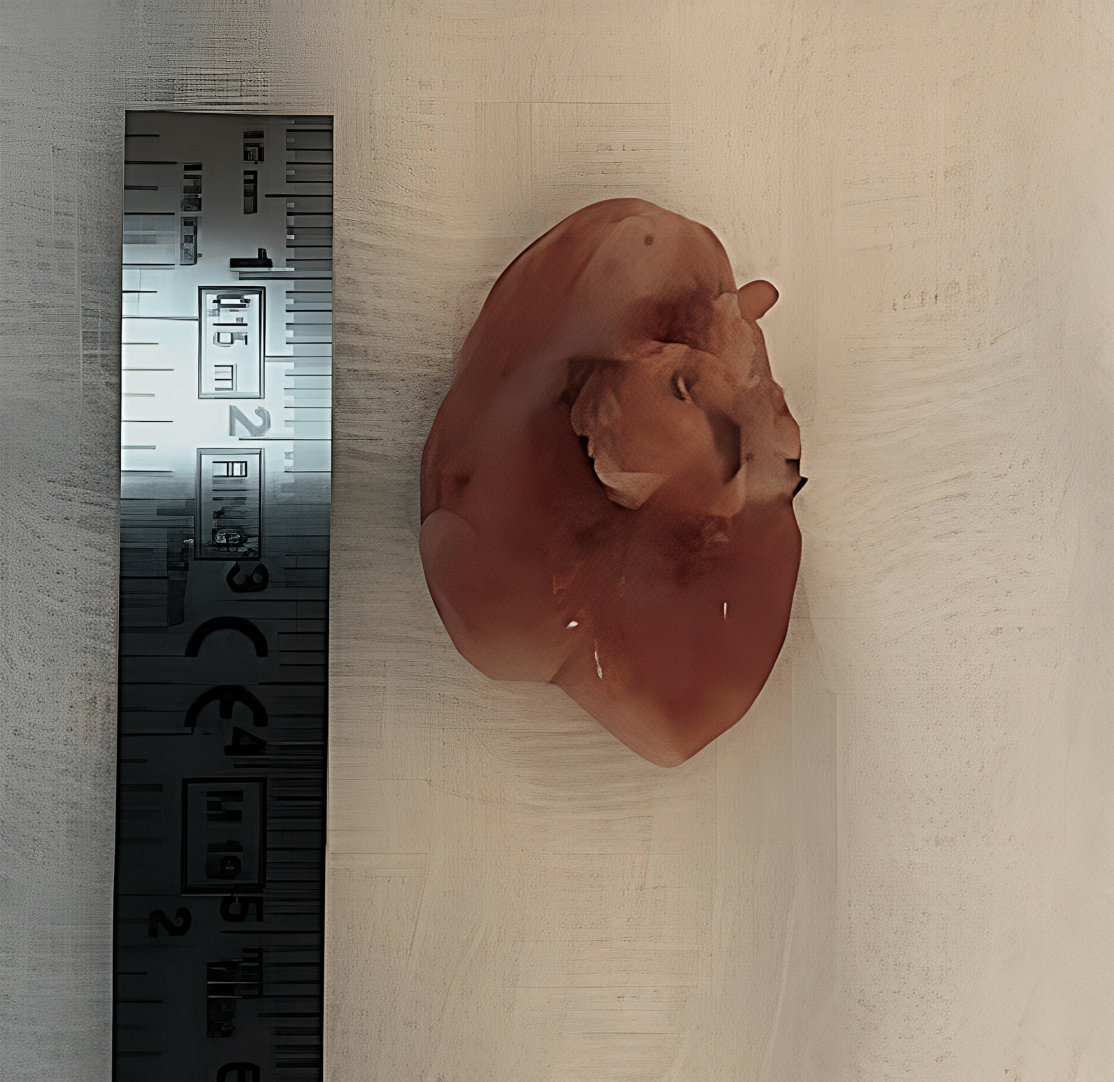
Post-excisional biopsy of the lesion

**Figure 7:**
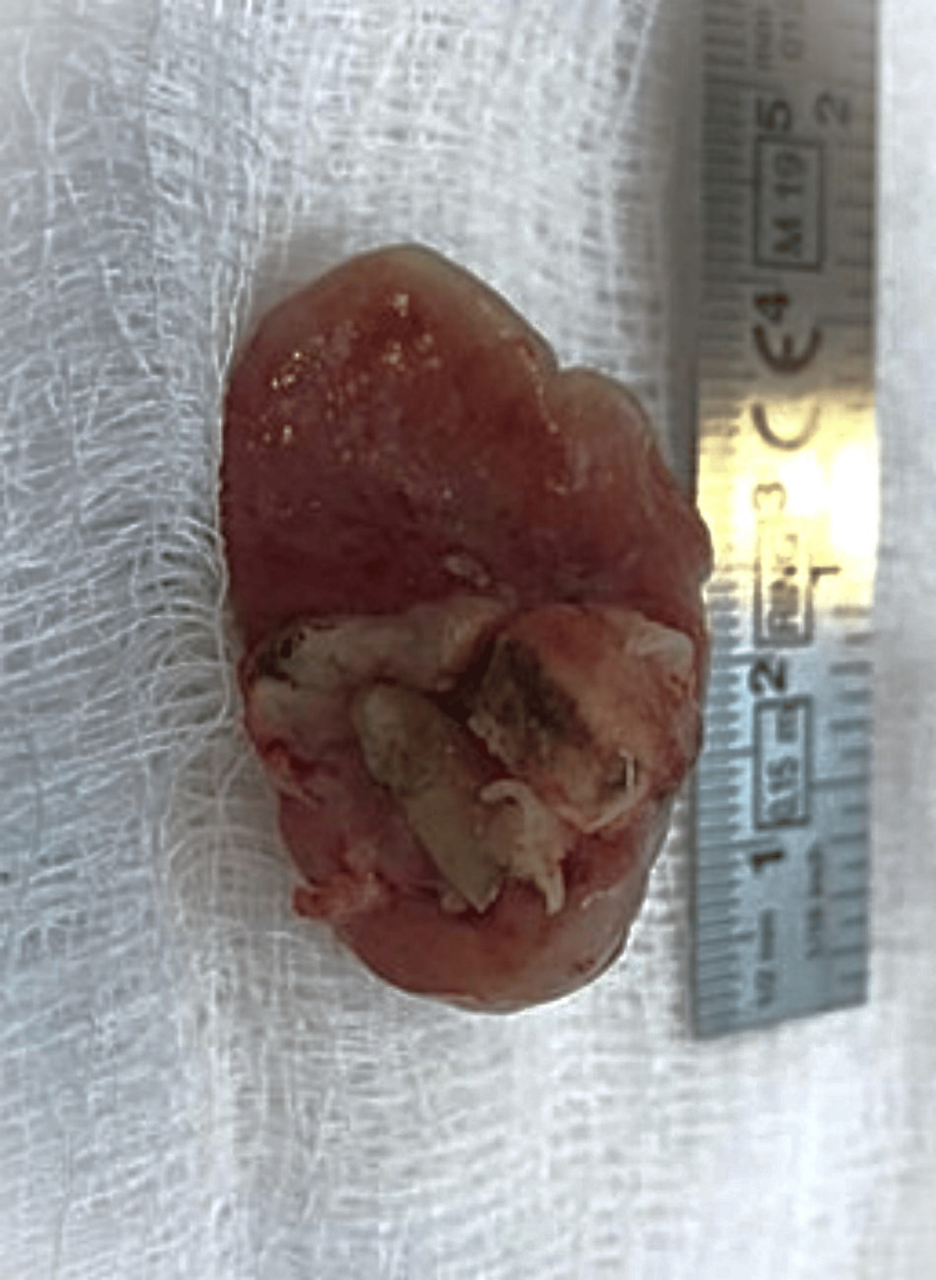
Post-excisional biopsy showing the remaining root attached to the lesion

The excisional biopsy procedure was undertaken, with the excised tissue being collected and submitted to the histopathology lab. The specimen was preserved in 10% formalin for the precise examination leading to the final diagnosis of GF. The excision of the lesion was skillfully conducted under local anesthesia, with the extraction of the remaining root of tooth number 27. The area of the lesion was curettage thoroughly, followed by wound closure using simple stitches employing 4-0 absorbable vicryl sutures (Figure 6 and Figure 7).

## Discussion

The case report and other results in various studies [2,6,10] provide valuable insights into GFs' epidemiological and clinical characteristics, shedding light on their distribution, gender, and clinical presentations. GFs represent fibrous growths within the gingival tissue that exhibit ambiguity in existing literature.

Numerous lesions within the oral mucosa exhibit a reactive nature. The gingiva being consistently exposed to various irritants such as plaque, calculus, food impaction, faulty restorations, broken down roots, and forceful brushing are particularly vulnerable. Prolonged irritation can trigger persistent inflammation, thereby promoting the proliferation of endothelial cells and chronic inflammatory cells [14].

The studies' findings indicate that GFs predominantly affect females, constituting 90% of the cases. The mean age of 45.11 years in a study by Bawazir et al. reflects a broad range, emphasizing that this lesion can manifest across various life stages. Regarding anatomical distribution, the study demonstrates a preference for the maxillary gingiva, with approximately 62% of cases reported in this region. The anterior incisor regions are notably more prone to these fibromas, accounting for 66.7% of cases, followed by canine and first premolar regions.

Gingival enlargements encountered in the oral cavity primarily arise from reactive hyperplasia rather than neoplastic origins, posing a diagnostic challenge for clinicians [15]. GF shares similarities with POF; both are identified as manifestations of cellular proliferation within a dense fibrous matrix. However, a distinguishing feature of POFs is the presence of calcification foci within their structure, setting them apart from GF [16].

In a case presented by Jaju et al., a well-defined, pink-colored gingival growth of 15×10 mm was observed, situated between the mesial line angle of tooth number 11 and the distal line angle of tooth number 12. Comprehensive scaling and root planing were performed, accompanied by detailed oral hygiene instructions. Surgical growth excision was carried out under local anesthesia, which was done using electrocautery. The patient was provided with ibuprofen for pain relief [17]. 

In another case, a 38-year-old female patient who had previously had her upper right 7 tooth extracted due to similar problems presented with mobility, dull pain, and swelling. Gingival plasmacytosis was discovered after an excisional biopsy, which was distinguished by pronounced squamous hyperplasia, a subepithelial plasmacytic infiltrate without atypia, and a unique plasma cell morphology. The significance of a thorough examination and histopathological analysis in dental cases with unusual presentations is underlined by this diagnosis.

Squamous papillomas, benign lesions that are frequently found in the oral mucosa, particularly on the palate and lips, have been studied in another case study. The lesions were slow-growing, painless papules with distinctive characteristics. In particular, the existence of comparable lesions on the hands raises the possibility of auto-inoculation [17]. Another case draws attention to squamous papillomas, common benign lesions of the oral mucosa that mainly affect the lips and palate. Consideration of auto-inoculation is prompted by the painless, slow-growing papules with distinctive features, especially when hand lesions are present at the same time. This emphasizes how crucial it is to thoroughly evaluate patients and educate them about potential self-transmission [18].

## Conclusions

GFs represent a distinct subset of oral fibrous lesions and gingival overgrowth with a diverse clinical profile. Their wide age distribution, gender predilection towards females, and clinical variables underscore the need for further research to elucidate their etiological underpinnings and develop more accurate diagnostic criteria. Collaborative efforts between clinicians, researchers, and pathologists are pivotal in advancing our knowledge, refining treatment strategies, and ultimately improving the quality of care for individuals affected by GFs. We added a case of GF to the literature, a newly recognized oral lesion that may need further clinicopathologic studies.
